# Physical and psychological workloads and their association with occupational fatigue among hospital service personnel

**DOI:** 10.1186/s12913-022-08530-0

**Published:** 2022-09-12

**Authors:** Morteza Ahmadi, Alireza Choobineh, Ali Mousavizadeh, Hadi Daneshmandi

**Affiliations:** 1 Department of Occupational Health and Safety Engineering, School of Health, Shiraz University of Medical Sciences, Shiraz, Iran; 2 Research Center for Health Sciences, Institute of Health, Shiraz University of Medical Sciences, Shiraz, Iran; 3 Department of Biostatistics and Epidemiology, School of Health, Yasuj University of Medical Sciences, Yasuj, Iran

**Keywords:** Hospital service personnel, Iran, Occupational fatigue, Physical workload, Psychological workload

## Abstract

**Background:**

Physical and psychological workloads are a vital issue in the workplace. This study aimed to investigate the association between physical and psychological workloads and occupational fatigue among Iranian hospital service personnel. In Iran, hospital service personnel refers to a group of healthcare workers who undertake a range of duties, such as moving and carrying the hospital waste, transporting patients by wheelchair or gurney to the operating room, x-ray department, other wards, or other locations around the facility, performing cleaning tasks such as changing linens, mopping floors, and sterilizing equipment, and following infection control procedures to reduce the risk of spreading germs within the hospital setting.

**Methods:**

This cross-sectional study was conducted on 198 Iranian hospital service personnel. The response rate was 86%. The data were gathered using 1) The Persian version of the Job Content Questionnaire (P-JCQ) for assessing physical and psychosocial workloads and 2) The Persian version of the Swedish Occupational Fatigue Inventory (P-SOFI-20) for assessing fatigue dimensions.

**Results:**

According to the P-JCQ, the physical and psychological workload intensities were high in 72.7% and 47% of the participants, respectively. Based on the P-SOFI, the participants’ mean scores of “physical fatigue” (21.73 ± 6.2), “psychological fatigue” (13.61 ± 5.76), and “fatigue due to shift work” (18.17 ± 5.6) were moderate, while the mean score of “general fatigue” was high (27.3 ± 6.98). The findings revealed that various types of fatigue are associated with age, gender, marital status, daily working hours, and psychological workload.

**Conclusions:**

Psychological workload was a determinant of occupational fatigue among Iranian hospital service personnel. Hence, an interventional program, including job enrichment, job rotation, and work-rest cycle, is recommended.

## Introduction

Workload is one of the most critical stressors in the workplace. It refers to the amount of physical or mental work performed by workers in a given time frame [1]. Physical workload includes placing the body in a specific posture or condition. The ability to perform physical work depends on age, sex, type of physical activity, environmental factors, heart rate, and body weight [2, 3]. Psychological workload has a multidimensional and complex structure affected by the environment, organizational factors, and psychological and cognitive abilities [4]. Designing ergonomic workstations and proper personnel selection for different occupations increase the workforce’s physical and mental abilities to perform a task [5, 6]. If employees’ physical and mental capabilities do not conform to their job requirements, it will lead to health and safety problems, productivity loss, and increased medical costs [7].

The previous studies have reported a high level of physical and psychological stress and a high workload among healthcare personnel [8–11]. Workload was among the most stressful and influential factors on service personnel’s behavior and efficiency [12]. Service personnel’s workload was associated with their physical and psychological duties and cognitive and mental tasks [13]. In therapeutic environments, such factors as high speed, high volume of work, and lack of social support could increase workload [14]. High workload, in turn, was one of the reasons for errors among service staff in treatment wards, because it caused them to have insufficient time to carry out their caretaking responsibilities. High workload has been mentioned to affect the quality of service delivery to patients by hospital staff [15, 16]. Evaluation of service personnel’s abilities in terms of costs is vital for employers, and improving them can increase human resources productivity in industries and organizations [17]. Therefore, workload assessment among hospital service personnel is essential to ensure the provision of desired services for patients [18].

Fatigue is a common phenomenon influenced by individual and environmental factors. Due to its adverse effects on health, safety, human productivity, and efficiency, fatigue must be monitored among service personnel. A tired person does not have the power and motivation to perform mental and physical tasks, is slow, and has inefficient performance [19]. Fatigue in workplaces stems from the lack of balance among effort, work duration, and rest time [20]. The negative consequences of occupational fatigue include increased human error, memory damage, decreased decision-making and reasoning power, increased risk of depression and anxiety, reduced efficiency, and disability. Therefore, fatigue leads to lower job performance and reduces the ability to perform physical and mental work [21–26].

Hospital service personnel, similar to other occupations, are at risk of work-related injuries due to fatigue, and the number of these injuries is equal to or even more than those in high-risk occupations [27]. Thus, this job group has been ranked sixth among the top ten jobs with a high risk of injury [28].

In Iran, hospital service personnel refers to a group of healthcare workers who undertake a range of duties, such as moving and carrying the hospital waste, transporting patients by wheelchair or gurney to the operating room, x-ray department, other wards, or other locations around the facility, performing cleaning tasks such as changing linens, mopping floors, and sterilizing equipment, and following infection control procedures to reduce the risk of spreading germs within the hospital setting. These duties include physically (e.g., prolonged static/awkward postures, extra force exertion, repetitive motions, manual material handling, and contact stress) and psychosocially (e.g., high demands and low job control or social support) demanding tasks [29]. In this context, a previous study reported that about 20% of hospital service personnel belonged to the high category of psychological workload [29].

The current study aimed to assess the association between physical and psychological workloads and occupational fatigue among Iranian hospital service personnel.

## Methods

This cross-sectional study was performed on Iranian hospital service personnel affiliated to Yasuj University of Medical Sciences (YUMS), Iran. Totally, 198 full-time service personnel with at least one year of work experience were selected after being provided with oral information about the study objectives and protocol. In this study, the response rate was 86%.

The nature of work and working conditions of the participants were almost similar, and attempts were made to avoid confounding variables that would distort the findings. Subjects were selected by census sampling method from the general wards of 3 hospitals of YUMS. Individuals who worked in hospital wards with different nature of work did not include in the study.

The study was approved by the Ethics Committee of Shiraz University of Medical Sciences (SUMS). Additionally, the study was performed in accordance with the Helsinki Declaration of 2013 [30].

At first, the study was explained orally to potential subjects, and all pertinent information, such as purpose, procedures, risks, benefits, and alternatives to participation was provided. All subjects were allowed an ample opportunity to ask questions. Those hospital service personnel willing to cooperate in this study signed a written informed consent form and were enrolled. Then, the participants completed the paper version questionnaires described below through self-reporting at their workplace during their work shift. After collecting the data from each subject, the questionnaires were checked by the researchers. If important data had not been inserted, the participant was asked to present any questions or ambiguities and after receiving an explanation, enter the missing information.

### Data collection

The data were collected using the following instruments:

#### Demographic/occupational questionnaire

This questionnaire included questions about age (years), height (cm), weight (kg), work experience (years): It means the length of time that a person has been working as a hospital service personnel in the affiliated hospital, working hours per day, sex (male and female), marital status (single and married), number of children, and education level (diploma and lower, associate degree, and bachelor’s and higher degrees).

#### The Persian version of the Job Content Questionnaire (P-JCQ)

This questionnaire was used to assess the service personnel’s perception and judgment about the workplace’s physical and psychological workloads. In this questionnaire, the physical and psychological workloads were measured using 12 and 8 items, respectively. The answer to each item was scored on a four-point scale (strongly agree to strongly disagree). For physical workload, the responses were dichotomized (1 and 2 versus 3 and 4). They were summed up as a new variable called “total physical workload”, the value of which varied from 0 to 12. Then, based on the total score of physical workload, the participants were classified into three groups; i.e., low (0 to 2), average (3 to 9), and high (10 to 12) physical workload. For psychological workload, the responses were obtained as two states (1 and 2 versus 3 and 4) and then, they were summed up as a new variable called “total psychological workload”, the value of which varied from zero to eight. Based on the total psychological workload score, the participants were classified into two groups, namely low (0 to 5) and high (6 to 8) psychological workload. The psychometric properties of the P-JCQ have been reviewed and approved by the Cronbach’s alpha coefficient of 0.82 [31].

#### The Persian version of the Swedish Occupational Fatigue Inventory (P-SOFI)

The SOFI-20 consisted of 20 items answered using an 11-point numerical rating scale (0 = not at all and 10 = to a very high degree). The items were categorized into five dimensions, including “lack of energy”, “physical exertion”, “physical discomfort”, “lack of motivation”, and “sleepiness”. The scores of each dimension could range from a minimum of 0 to a maximum of 40. Then, four types of fatigue are calculated:

- a) Physical fatigue: This type of fatigue is calculated by the mean score of the “physical exertion”, and “physical discomfort” dimensions.
- b) Psychological fatigue: This type of fatigue is calculated by the mean score of the “lack of motivation” and “sleepiness” dimensions.
- c) Fatigue due to shift work: This type of fatigue is calculated by the mean score of the “sleepiness”, “lack of energy”, and “lack of motivation” dimensions.
- d) General fatigue: The score of general fatigue is the mean score of the “lack of energy” dimension items.

Based on the SOFI-20 users’ guide, the score of each dimension was rated based on severity; i.e., low (mean score < 8.5), medium (8.5 < mean score < 23.5), and high (mean score > 23.5) levels of fatigue, based on the quartiles of scores distribution [32, 33]. The psychometric properties of P-SOFI-20 were confirmed by Javadpour et al. [34].

### Data analysis

The Statistical Package for Social Sciences 16 (SPSS Inc., Chicago, IL, USA) was used to analyze the data. At first, Kolmogorov–Smirnov and Shapiro–Wilk tests were used to test the normality of the data. Descriptive statistics such as frequency, percentage, and mean ± standard deviation were used for the description of demographic/occupational details (Table 1), physical and psychological workloads frequencies (Table 2), and the frequency of fatigue types derived from the P-SOFI (Table 3). A multiple linear regression model was used for evaluating relationships between P-SOFI subscales (physical fatigue, psychological fatigue, fatigue due to shift work, general fatigue) as continuous targets and potential predictors, including age, daily working hours, work experience, gender, marital status, number of children, education level, and physical and psychological workloads. *P* < 0.05 was considered statistically significant.

**Table 1:** Demographic/occupational details of the studied hospital service personnel (*n* = 198)

Quantitative variables	M ± SD	Min	Max
**Age (years)**	39.53 ± 9.17	21	70
**Height (cm)**	169.40 ± 9.82	144	200
**Weight (kg)**	73.45 ± 11.94	48	115
**Daily working hours**	15.88 ± 7.58	6	24
**Work experience (years)**^a^	12.14 ± 8.66	1	32
**Quantitative variables**		**No**	**%**
**Gender**	Male	66	33.3
	Female	132	66.7
**Marital status**	Single	27	13.6
	Married	171	86.4
**Number of children**	≤ 1	73	36.9
	2	55	27.8
	3	35	17.7
	4	15	7.6
	≥ 5	20	10
**Education level**	Diploma and lower	148	74.7
	Associate degree	24	12.1
	Bachelor’s and higher degrees	26	13.1

^a^It means the length of time that a person has been working as a hospital service personnel in the affiliated hospital

**Table 2:** Physical and psychological workloads among the participants based on the P-JCQ (*n* = 198)

Workload	No	%
**Physical**		
Low (0–2)	-	-
Moderate (3–9)	54	27.3
High (10–12)	144	72.7
**Psychological**		
Low (0–5)	105	53
High (6–8)	93	47

**Table 3:** The frequency of fatigue types derived from the P-SOFI (*n* = 198)

Types of fatigue	Low (< 8.5)		Moderate (8.5 to 23.5)		High (> 23.5)	
	**No**	**%**	**No**	**%**	**No**	**%**
**Physical fatigue**	8	4	130	65.7	60	30.3
**Psychological fatigue**	38	19.2	151	76.3	9	4.5
**Fatigue due to shift work**	10	5.1	160	80.8	28	14.1
**General fatigue**	6	3	32	16.2	160	80.8

## Results

Some demographic/occupational details of the studied hospital service personnel have been presented in Table 1.

### Physical and psychological workloads assessed by the P-JCQ

The data related to the physical and psychological workloads derived from the P-JCQ have been presented in Table 2. Accordingly, 27.3% and 72.7% of the participants perceived physical workload with moderate and high intensity, respectively. In addition, the intensity of psychological workload was low in 53% and high in 47% of the participants. The means ± standard deviations of physical and psychological workloads were 10.56 ± 2.05 and 5.53 ± 1.19, respectively.

### Occupational fatigue assessed by the P-SOFI

Based on the P-SOFI, 30.3%, 4.5%, 14.1%, and 80.8% of the participants had severe “physical fatigue”, “psychological fatigue”, “fatigue due to shift work”, and “general fatigue”, respectively (Table 3). Besides, the means ± standard deviations of “physical fatigue”, “psychological fatigue”, “fatigue due to shift work”, and “general fatigue” were 21.73 ± 6.20, 13.61 ± 5.76, 18.17 ± 5.62, and 27.30 ± 6.98, respectively.

### Potential factors associated with various types of the occupational fatigue

Table 4 shows the association between fatigue derived from the P-SOFI and physical and psychological workloads based on the P-JCQ. As shown, various types of fatigue are associated with age, gender, marital status, daily working hours, and psychological workload (*p* < 0.05).

**Table 4:** Associations between fatigue derived from the P-SOFI and physical and psychological workloads based on the P-JCQ (*N* = 198)

**P-SOFI subscale**	**Independent variable**	**Beta**	**95% CI**	***p***^a^
**Physical fatigue**	Age	1.79	1.31–2.51	< 0.001
	Daily working hours	1.16	1.05–2.37	< 0.001
	Psychological workload	1.91	1.07–2.79	< 0.001
**Psychological fatigue**	Age	1.67	1.13–2.16	0.008
	Female gender	1.24	1.07–2.05	0.002
	Marital status (single)	1.17	1.05–1.93	0.026
	Psychological workload	2.09	1.26–2.97	0.020
**Fatigue due to shift work**	Age	1.12	1.03–1.88	0.005
	Female gender	1.53	1.04–1.97	< 0.001
	Psychological workload	1.77	1.10–2.55	0.010
**General fatigue**	Age	1.36	1.13–2.27	0.010
	Daily working hours	1.46	1.19–2.11	< 0.001
	Female gender	1.48	1.06–1.97	0.028
	Psychological workload	1.73	1.13–2.23	< 0.001

^a^Multiple linear regression analysis of fatigue derived from the P-SOFI and workloads based on the P-JCQ adjusted for age, daily working hours, work experience, gender, marital status, number of children, education level. Only the statistically significant correlations are presented

Note: *p* = *p*-value, Multiple linear regression (*r*^2^ = 0.56, *p* = 0.02)

## Discussion

This study aimed to assess the association between physical and psychological workloads and occupational fatigue among Iranian hospital service personnel. The findings revealed no significant relationship between physical workload and occupational fatigue. On the other hand, there was a statistically significant association between psychological workload and various occupational fatigue types, including “physical fatigue”, “psychological fatigue”, “fatigue due to shiftwork”, and “general fatigue”.

### Physical and psychological workloads assessed by the P-JCQ

The current study results showed that 27.3% and 72.7% of the participants perceived physical workload with moderate and high intensity, respectively. In addition, the intensity of psychological workload was reported to be low in 53% and high in 47% of the participants. The means ± standard deviations of the physical and psychological workloads were 10.56 ± 2.05 and 5.53 ± 1.19, respectively. These findings were not consistent with those obtained by Choobineh et al. [8]. Some studies have shown that most participants perceived a high mental workload [10, 11, 35–39], while most of the hospital service personnel in the current study (53%) perceived a low level of psychological workload. Salmani Nodooshan et al. also reported that 83.4% of the hospital attendants belonged to the low category of psychological workload [29]. These differences could be attributed to the population under investigation, type of physical workload, type of work environment, difference in job responsibilities, and different assessment tools.

### Occupational fatigue assessed by the P-SOFI

The findings of the present study indicated that the means ± standard deviations of “physical fatigue”, “psychological fatigue”, “fatigue due to shift work”, and “general fatigue” were 21.73 ± 6.20, 13.61 ± 5.76, 18.17 ± 5.62, and 27.30 ± 6.98, respectively. These findings were in agreement with those of the study conducted by Asadi et al. [40] and the Cochran study, which demonstrated moderate chronic fatigue among the participants [41]. The results were also consistent with those of a study performed using the Seman-Perlie Questionnaire [42], and research carried out by Azmoon et al. using the Multidimensional Fatigue Inventory (MFI) [43]. In contrast, Choobineh et al. [31] and Batak et al. (using the Piper Fatigue Scale (PFS)) [44] reported high levels of fatigue.

### Potential factors associated with various types of the occupational fatigue

Our findings revealed that various types of fatigue are positively associated with age, female gender, unmarried status, daily working hours, and psychological workload.

In this context, it has been revealed that occupational fatigue is associated with age [45] and female gender [46–48]. Dochi et al. in their study verified that unmarried status was more associated with fatigue [45, 49]. In addition, the literature suggests that there is an association between occupational fatigue and working long hours [50].

Although the hospital service personnel were involved in physical tasks, no significant relationships were found between physical workload and fatigue in the current study. This finding was in line with that of the research conducted by Salmani Nodooshan et al. [29], but was contrary to those of some other studies [51, 52]. This difference could be attributed to different data gathering tools and work environments. In addition, the participants might not have answered the questions honestly because of being afraid of losing their jobs. The society’s economic status and the participants’ education levels might have played a role (almost 87% of the participants had below bachelor’s degrees).

On the other hand, the current study results showed a significant correlation between psychological workload based on the P-JCQ and various types of fatigue derived from the P-SOFI, which was inconsistent with the study results performed by Arellano et al. [53]. However, Motamedzade et al. [54] and Fan et al. [55] reported a significant association between psychological workload and occupational fatigue.

Although at first glance, it seems that the fatigue of the studied subjects is more influenced by physical workload, the findings of our study demonstrated that their fatigue is more influenced by psychological workload. In this context, we found that job demands such as time pressure can lead to a high psychological workload. According to psychological job demands, hospital service personnel are under pressure and at risk of job fatigue. On the other hand, it was revealed that this working group has little control over various aspects of their jobs. In this context, the findings of a meta-analysis study showed that high psychological workload was consistently related to depressive symptoms, mental disorders, and employees’ intention to leave the job [56].

Based on our findings, paying attention to the psychological workload as a predictor of hospital service personnel fatigue has special importance and place. So, in this context, there are suggestions for reducing physical/psychological workloads and as well fatigue among hospital service personnel:

− Ensuring that the physical/ psychological workloads are in line with workers’ capabilities and resources.
− Organizational support could reduce work environment stressors to relieve physical/psychological workloads and fatigue.
− Allowing for recovery from demanding tasks could increase control over work pace.
− Designing jobs to provide meaning, stimulation, and opportunities for hospital service personnel to use their skills.
− Clearly defining hospital service personnel roles and responsibilities.
− Giving hospital service personnel opportunities to participate in decisions and actions affecting their jobs.
− Encouraging work-life balance through family-friendly benefits and policies.

### Limitations

Given the cross-sectional design of the study and data collection by self-report, the findings should be interpreted cautiously. Moreover, this study was performed among hospital service personnel in Yasuj. Therefore, the results might not be generalized to other working groups. Furthermore, this study was conducted on a small sample. Hence, a larger sample size is needed to achieve more robust results in this context.

## Conclusion

In this study, most participants experienced a high physical workload in their work environment. The “general fatigue” was the most significant perceived fatigue by the hospital services personnel. In addition, the psychological workload was one of the determinants of job fatigue perceived by the hospital service personnel. Thus, an interventional program, including job rotation between different hospital wards and work-rest cycle, is proposed to improve the physical and psychological workloads and reduce job fatigue among hospital service staff.

## Data Availability

We are unable to share data publicly because of ethical and legal restrictions. The datasets used and/or analysed during the current study are available from the corresponding author on reasonable request.

## Abbreviations

P-JCQ: Persian version of the Job Content Questionnaire
P-SOFI: Persian version of the Swedish Occupational Fatigue Inventory
MFI: Multidimensional Fatigue Inventory
PFS: Piper Fatigue Scale
